# Real-world evidence on migraine treatment in routine clinical practice: insights from the Türkiye migraine registry study using artificial intelligence algorithms

**DOI:** 10.3389/fneur.2026.1848551

**Published:** 2026-08-17

**Authors:** Pınar Yalınay Dikmen, Elif Ilgaz Aydinlar, Seda N. Dumlu, Tugay Onal, Salih Gumru, Efe Onganer, Alex Loleyt

**Affiliations:** 1 Department of Neurology, Acibadem Ali Aydinlar University School of Medicine, Istanbul, Türkiye; 2 Department of Computer Engineering, Faculty of Engineering and Natural Sciences, Acibadem Mehmet Ali Aydinlar University, Istanbul, Türkiye; 3 Pfizer Türkiye, Istanbul, Türkiye; 4 Department of Medical Sciences, Family Medicine, Acibadem Mehmet Ali Aydinlar University School of Medicine, Istanbul, Türkiye; 5 Traverse Health, Amsterdam, Netherlands

**Keywords:** artificial intelligence, electronic health records, migraine, natural language processing, real-world evidence, Türkiye

## Abstract

**Background:**

Despite available diagnostic and therapeutic options, a major unmet need persists in migraine care. This study applied artificial intelligence (AI) to extract structured insights from routine care and assess migraine burden, diagnosis, and treatment in Türkiye.

**Methods:**

The Türkiye Migraine Registry Study was a retrospective, observational study across 23 hospitals and clinics in eight cities. Anonymized electronic health records (EHRs) of 11,023 adults treated between January 2021 and October 2023 were analyzed. A validated AI framework with natural language processing and machine learning extracted demographics, comorbidities, imaging, and prescriptions. Descriptive statistics summarized outcomes.

**Results:**

Of 90,381 neurology patients, 16,751 (18.5%) had migraine or suspected migraine; 11,023 met inclusion. Only 46.9% carried an ICD-10 migraine code, while 53.1% were coded under other headache syndromes yet frequently received migraine-specific therapies. The cohort was 73% female, mean age 41.5. Neuroimaging was frequent (58.7% MRI, 15.2% CT), and 18.4% visited emergency care. Preventive therapy was prescribed to 63.7%, mainly antidepressants (31.4%) and beta-blockers (19.4%). OnabotulinumtoxinA and CGRP monoclonal antibodies were used in 10.3 and 3.4%.

**Conclusion:**

AI-assisted EHR analysis revealed underdiagnosis, high imaging use, and limited adoption of mechanism-specific preventives in Türkiye, underscoring the need for improved diagnostic accuracy and broader access to modern therapies.

## Introduction

Migraine is a highly prevalent and disabling primary headache disorder, affecting over one billion individuals worldwide. It imposes a substantial burden in terms of personal suffering, reduced productivity, and economic costs across healthcare systems (1, 2). Characterized by recurrent attacks of moderate to severe headache—often accompanied by nausea, photophobia, and phonophobia—migraine predominantly affects individuals between the ages of 25 and 55, typically the most productive years of life (3). Globally, its prevalence is estimated at 14–15%, with considerable regional variation, ranging from 9.3% in East Asia to over 30% in South Asia (4). In Türkiye, the reported prevalence is 16.4% (5).

Despite the availability of well-defined diagnostic criteria and a broad spectrum of acute and preventive treatment options, migraine care remains suboptimal on a global scale. Many patients remain undiagnosed or inadequately treated, and less than 15% of eligible individuals worldwide receive preventive therapy (1, 2). This highlights a persistent gap between evidence-based guidelines and real-world clinical practice—driven in part by the subjective nature of symptom reporting and the lack of reliable biomarkers in routine care (6).

Migraine is a common yet diagnostically challenging neurological disorder, making it particularly well-suited for real-world data analysis (7). In recent years, there has been increasing emphasis on leveraging real-world evidence (RWE) to inform clinical practice, regulatory frameworks, and health policy decisions (7–10). Accurate migraine diagnosis depends on recognizing a cluster of symptoms such as unilateral, pulsating pain of moderate to severe intensity, often aggravated by routine physical activity along with associated features such as nausea, photophobia, and phonophobia. However, these symptom details are frequently recorded in free-text clinical notes within Electronic Health Records (EHRs), limiting their accessibility through conventional analytic methods. To overcome this barrier, artificial intelligence (AI) technologies, particularly natural language processing (NLP) and machine learning (ML), have emerged as powerful tools for extracting structured insights from unstructured clinical data (9). When applied effectively, these AI-driven approaches can enhance data quality and generate robust evidence, supporting more informed decision-making by regulators, payers, clinicians, and patients alike (8–11).

To address this gap, the Türkiye Migraine Registry Study (TMRS) was launched as a large-scale, real-world observational initiative leveraging anonymized electronic health records (EHRs) from 23 hospitals and outpatient clinics across eight cities. The study employed a validated AI framework developed by Traverse Health, integrating NLP and ML techniques to extract structured information from unstructured clinical narratives. Its primary objective was to characterize the demographics, clinical profiles, and treatment patterns of adult patients with confirmed or suspected migraine in Türkiye.

## Methods

### Study design and data sources

**Figure 1:**
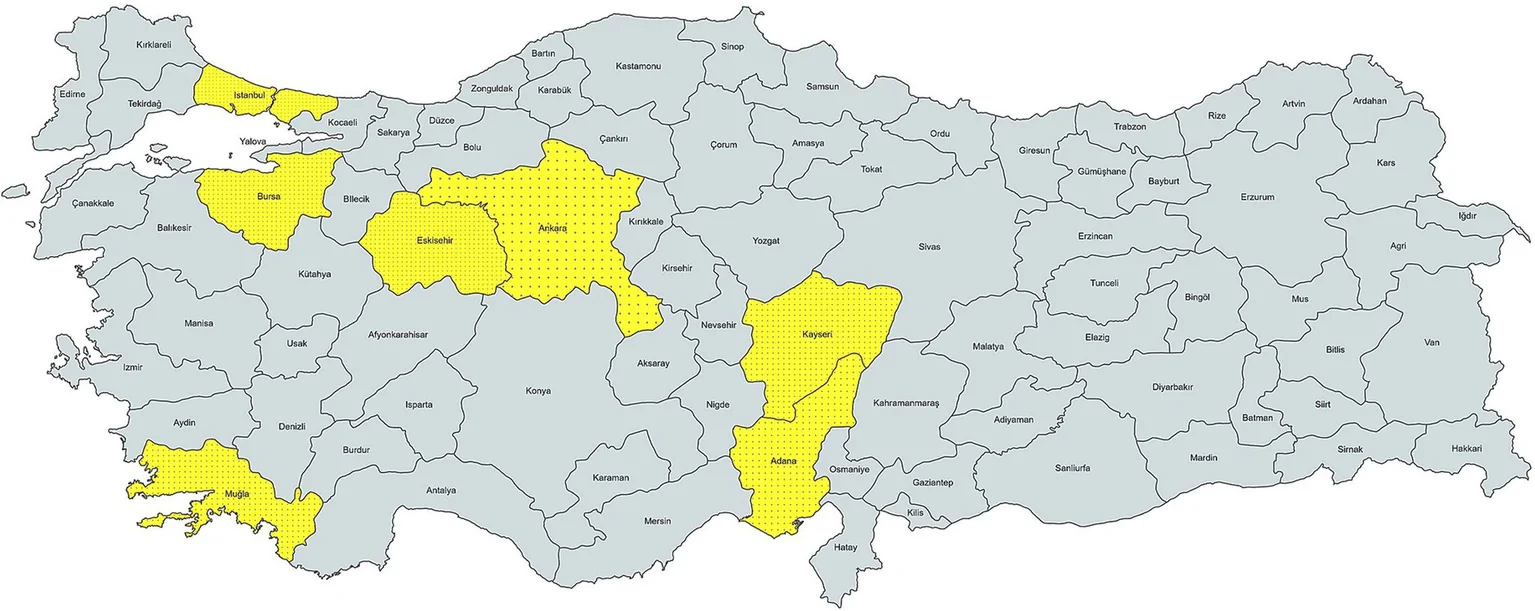
Location of Acıbadem hospitals and outpatient clinics. The dataset included both structured and unstructured clinical data collected from 23 hospitals and outpatient clinics across eight cities in Turkey. These locations comprised facilities in Adana, Ankara, Bursa, Eskişehir, Kayseri, Kocaeli, and Muğla (Bodrum and Bodrum TM), as well as multiple sites in Istanbul, including Alife KOS, Altunizade, Atakent, Ataşehir T. M., Bağdat, Bahçeşehir, Bakırköy, Beylikdüzü, Dr. Şinasi Can, Etiler, Fulya, Göktürk, International, Maslak, and Zekeriyaköy.

This non-interventional, retrospective study utilized anonymized EHRs from the Acibadem Health Group, a leading private hospital network in Türkiye (Figure 1). The analyzed electronic health records were contributed by 44 neurologists from the Acıbadem Hospital Neurological Study Group across the participating hospitals. The study was approved by the Acibadem University School of Medicine Ethics Committee and conducted in line with institutional guidelines (2023–21/719).

### Data details and patient overview

Between January 2021 and October 2023, a total of 21,273 patients diagnosed with migraine or suspected of having undiagnosed migraine (initially coded under other headache syndromes) received care at 23 hospitals and outpatient clinics affiliated with the Acibadem Health Group, spanning eight cities across Türkiye. For the purposes of this analysis, data from 11,023 patients were included—specifically those with available treatment records involving at least one medication from a predefined list of International Nonproprietary Names (INNs) for headache or migraine.

Following data acquisition, an extract-transform-load (ETL) process was conducted to standardize and integrate the data into the Traverse Health Common Data Model (CDM). This process involved raw data validation, duplicate removal, parsing of free-text entries using ML algorithms, harmonization and encoding of variables, resolution of missing data, and quality assurance testing. The entire ETL pipeline was documented in a dedicated technical specification: Acibadem Migraine—Traverse Health CDM Business Rules Specification (Supplementary material 1).

### Eligibility criteria

*Event 1 (Base Population)*: Identified patients whose primary diagnosis between January 2021 and October 2023 corresponded to migraine (International Classification of Diseases (ICD)-10 codes: G43.0–G43.9) or other primary and secondary headache disorders (ICD-10 codes: G44.0–G44.4, G44.8). While the G43 series specifically indicates migraine subtypes, the G44 series includes other headache syndromes such as cluster headache, tension-type headache, and medication-overuse headache.

*Event 2*: Age ≥ 18 years, during the same period.

*Event 3*: Patients treated with target INNs for acute or preventive treatment as per the list provided in the Methods section (section 2.3 of Supplementary material 2).

*Event 4*: Patients treated in specified departments (Neurology, Neurosurgery, Emergency Department, Child Neurology, Psychiatry, Algology).

### Data handling

The “all_diagnosis_codes_for_this_visit” column in the dataset contains one or more ICD-10 codes, which may include both main and concomitant diagnoses.

Drug information was captured in plain text. Free text parsing was used to extract drug names and other related information. Active ingredients and Anatomical Therapeutic Chemical (ATC) codes of the drugs were identified using internet scraping, mainly from ilacabak.com, a Turkish online database that provides detailed information on medications, including their composition, indications, and regulatory status.

**Figure 2:**
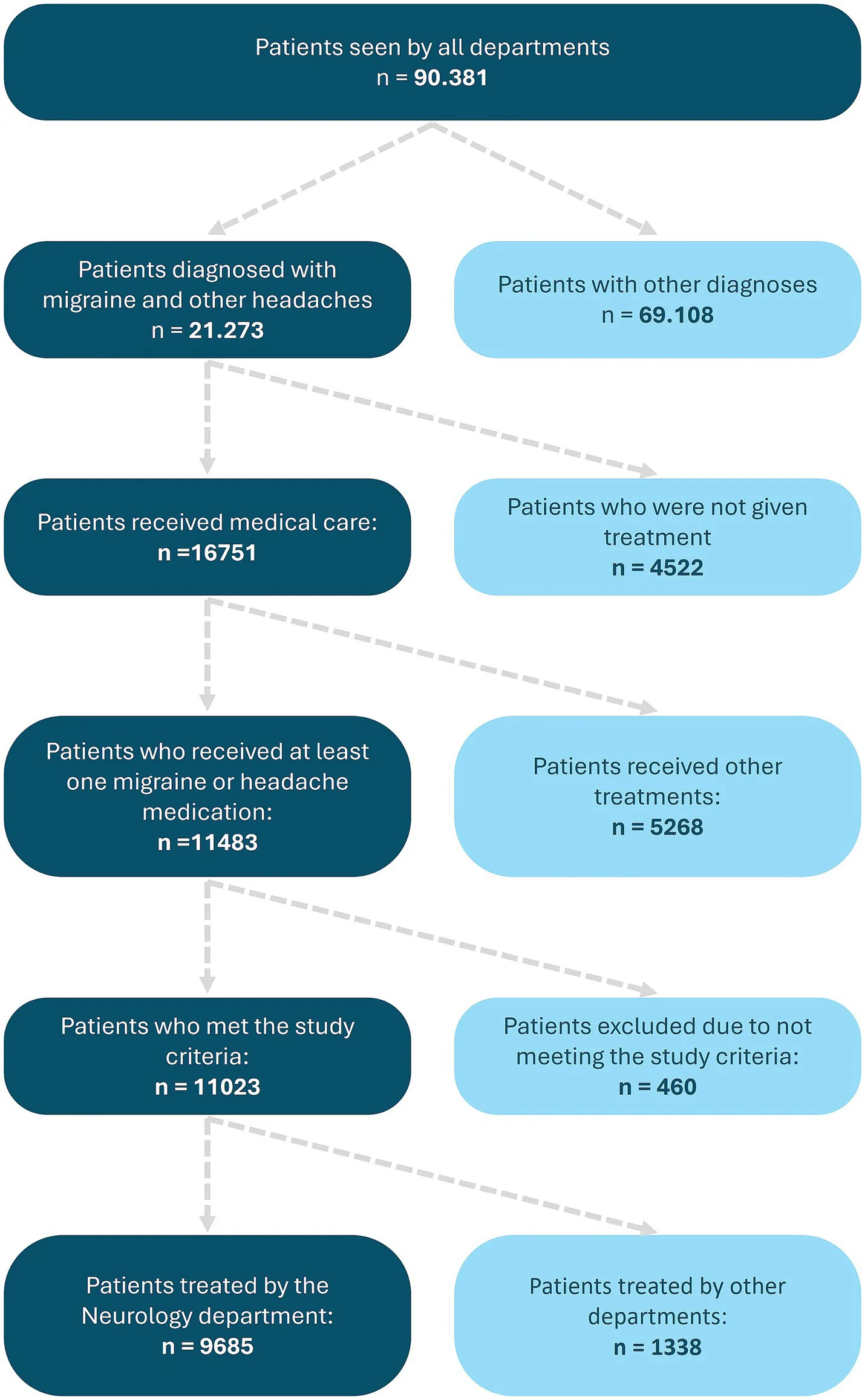
Patient funnel of the study.

*Sample Size*: Figure 2 shows patient funnel of the study. The study analyzed 21,273 patient records (Figure 2). The final dataset comprised 11,023 patients who met all criteria out of the original 21,273 analyzed. The criteria funnel illustrates the impact of each criterion on narrowing the number of eligible patients from the Base Population (patients diagnosed with migraine or other headache syndromes, aged 18 or older, of any gender). The order of criteria in the funnel is from most impact to least (Figure 2).

### Department distribution

Across all departments, 16,751 out of 21,273 patients (78.7%) diagnosed with migraine or other headache syndromes received medical care. Among them, 11,023 patients met the inclusion criteria for the study, and of these, 9,685 (87.9%) received treatment from a neurologist. Additionally, during the same period, a total of 90,381 adult patients received care for various diseases or conditions in the departments, including Neurology, Neurosurgery, Emergency Medicine, Child Neurology, Psychiatry, and Algology (across outpatient, inpatient, or emergency settings) of the 23 participating hospitals. Thus, patients with verified migraine or possible undiagnosed migraine (other headache syndromes) accounted for 18.5% (16,751/90,381) of all patients treated in the neurological departments.

### AI framework for migraine treatment analysis

To enable large-scale analysis of migraine-related information contained in electronic health records (EHRs), a dedicated artificial intelligence (AI) framework was developed by Traverse Health and integrated into the study workflow. The framework was designed to transform unstructured clinical narratives into structured variables suitable for analysis through a combination of natural language processing, named entity recognition (NER), rule-based information extraction, and data standardization.

Clinical information was extracted from multiple free-text EHR sources, including patient complaints, medical histories, physician notes, treatment records, follow-up documentation, and medication records. Before model development, all text underwent preprocessing procedures including cleaning, normalization, and concatenation of relevant clinical fields.

A manually annotated reference dataset consisting of 573 Turkish clinical free-text samples extracted from routine EHRs was created using the Prodigy annotation platform. The annotated dataset was developed to train the NER model and covered the major clinical concepts relevant to migraine documentation, including headache characteristics, migraine symptoms, triggers, neurological examination findings, medications, drug doses, treatment frequencies, symptom duration and intensity, and consultation-related information. Clinical entities were annotated according to a predefined annotation schema by trained annotators.

Automated information extraction was performed using a custom NER model developed through transfer learning from a pretrained multilingual transformer architecture (XLM-RoBERTa Large). Following entity recognition, rule-based relation extraction algorithms were applied to establish clinically meaningful relationships between extracted entities, including the linkage of headache frequency to headache descriptions and medication doses to their corresponding treatments or medication doses to specific treatments. The extracted information was subsequently standardized and mapped to the Traverse Health Common Data Model (CDM), enabling structured analyses across the study population.

Model development and evaluation were performed using the manually annotated reference dataset. Using this reference dataset, the core NER model achieved an F1 score exceeding 95% on the validation dataset. The performance of downstream extraction tasks varied according to the target variable. For symptom extraction, precision, recall, and F1-score were each 85.9%, whereas medication extraction achieved a precision of 97.5%, recall of 96.4%, and an F1-score of 97.0%.

To further improve the quality and consistency of extracted information, the AI framework incorporated multiple quality assurance procedures throughout the extraction pipeline. These included mapping extracted entities to standardized clinical terminology, manual verification of selected medication extraction outputs, independent review of selected clinical variables (including pain-location data), rule-based post-processing and consistency checks before incorporation into the final analytical dataset.

The role of the AI framework was not to generate diagnoses or clinical interpretations, but rather to identify, extract, and structure information already documented by clinicians during routine practice. Human experts defined the annotation schema, supervised model development, reviewed selected extraction outputs, and established quality-control procedures. The resulting structured dataset served as the basis for all subsequent analyses of patient characteristics, symptom profiles, healthcare utilization, and treatment patterns. Performance metrics of the AI framework are summarized in Supplementary Table S1.

### Statistical analysis

The study population comprised 11,023 patients meeting the inclusion criteria, defined as the Full Analysis Set (FAS). Patients were further stratified by index date/event, gender, age group, primary diagnosis, migraine subtype, treatment regimen, and healthcare facility. Descriptive statistics were used to summarize patient characteristics, medication use, and healthcare resource utilization. Categorical variables were reported as frequencies and percentages, while continuous variables were summarized using means, standard deviations, medians, and ranges. The detailed statistical methodology is outlined in the *Statistical Analysis Plan* (Supplementary material 2).

Missing data were expected because the study was based on routinely collected EHRs. No imputation procedures were performed. Analyses for each variable were conducted using available-case analysis, and denominators are reported where appropriate.

## Results

### Demographic and baseline characteristics

The distribution of data across the years (2021–2023) during the analyzed period was relatively consistent: Q1–Q4 2021 included 4,493 patients, Q1-Q4 2022 included 4,119 patients, and Q1–Q3 2023 included 2,411 patients. The majority of patients included in the analysis (comprising 10% or more of the FAS) received medical care in the provinces of Istanbul (35.1%), Kayseri (18.1%), Kocaeli (11.5%), Muğla (11.1%), and Eskişehir (10.3%).

The study included a total of 11,023 participants with a mean age of 41.5 ± 14 years and a median age of 39 years (range: 18–94) (Table 1). The age distribution was as follows: 18–29 years (18.2%), 30–39 years (31.7%), 40–49 years (26.1%), 50–59 years (11.7%), 60–64 years (3.7%), and ≥65 years (8.6%). The majority of the participants were female (73.0%), while 27.0% were male. Information on alcohol or tobacco use was available for 84.1% of the participants. Information on alcohol and/or tobacco use was available for 9,274 patients (84.1%). Overall, documented alcohol and/or tobacco use was present in 2,819 patients (25.6% of the total cohort), whereas no documented use was identified in 6,455 patients (58.6%).

**Table 1:** Baseline characteristics of the study population (full analysis set, *N* = 11,023).

Characteristic	Value
Number of patients	11,023
Age, years	
Mean ± SD	41.5 ± 14.0
Median (range)	39 (18–94)
Age group, *n* (%)	
18–29 years	2,006 (18.2%)
30–39 years	3,494 (31.7%)
40–49 years	2,877 (26.1%)
50–59 years	1,290 (11.7%)
60–64 years	408 (3.7%)
≥65 years	948 (8.6%)
Sex, *n* (%)	
Female	8,047 (73.0%)
Male	2,976 (27.0%)
Geographic distribution, *n* (%)	
İstanbul	3,869 (35.1%)
Kayseri	1,995 (18.1%)
Kocaeli	1,267 (11.5%)
Muğla	1,223 (11.1%)
Eskişehir	1,135 (10.3%)
Other provinces	1,534 (13.9%)
Alcohol and/or tobacco use	
Available records	9,274 (84.1%)
Documented alcohol and/or tobacco use	2,819 (25.6%)
No documented alcohol and/or tobacco use	6,455 (58.6%)

N, Number of subjects; SD, Standard deviation.

### Main disease diagnosis

A summary of headache diagnosis for the overall population (FAS) is presented in Table 2. Among the 11,023 patients in the study, 5,172 (46.9%) had a verified diagnosis of migraine. Among those with migraine, 40.0% had unspecified migraine, 35.8% had migraine without aura, and 4.6% had migraine with aura. Monthly headache days were reported in 17.2% of the migraine group, with a mean of 10.8 days and with a median frequency of 8 days per month (range: 1–30 days). Migraine attacks lasted longer in females (*n* = 460/664) (mean: 57.4 h) compared to males (*n* = 204/664) (38.1 h).

**Table 2:** Headache diagnoses in the cohort.

Main disease type	Subgroups/parameters statistics	Total (*N* = 11,023)
Patients with verified diagnosis of migraine, ICD-10 block: G43	Any migraine, *n* (%)	5,172 (46.9%)
	Migraine, unspecified (G43.9), *n* (%)	2,070 (18.8%)
	Migraine without aura (G43.0), *n* (%)	1,854 (16.8%)
	Other migraine (G43.8), *n* (%)	702 (6.4%)
	Migraine with aura (G43.1), *n* (%)	512 (4.6%)
	Complicated migraine (G43.3), *n* (%)	22 (0.2%)
	Status migrainosus (G43.2), *n* (%)	12 (0.1%)
	Number of patients with available data, *n* (%)	1894 (17.2%)
	Headache frequency (days per month) Mean± SD	10.8 ± 9.8
	Headache frequency (days per month) Median (min–max)	8.0 (1–30)
Patients with other headache syndromes (suspected* migraine), ICD-10 block: G44 or R51	Any other headache, *n* (%)	5,851 (53.1%)
	Tension-type headache (G44.2), *n* (%)	2,385 (21.6%)
	Other specified headache syndromes (G44.8), *n* (%)	2,230 (20.2%)
	Vascular headache, not elsewhere classified (G44.1), *n* (%)	1,062 (9.6%)
	Cluster headache syndrome (G44.0), *n* (%)	153 (1.4%)
	Drug-induced headache, not elsewhere classified (G44.4), *n* (%)	18 (0.2%)
	Chronic post-traumatic headache (G44.3), *n* (%)	3 (0.03%)
	Other headache syndromes, unspecified (G44), *n* (%)	0 (0%)
	Headache (R51), *n* (%)	0 (0%)
	Headache frequency (days per month)	
	Number of patients with available data, *n* (%)	1,381 (12.5%)
	Headache frequency (days per month) Mean± SD	12.7 ± 11.3
	Headache frequency (days per month) Median (min–max)	8.0 (1–30)

*A hypothetical assumption, not based on any criteria other than the presence of a headache. SD, Standard Deviation.

Additionally, a subgroup of patients with other headache syndromes was identified, presumed to include individuals with undiagnosed or suspected migraine. A total of 5,851 (53.1%) patients were diagnosed with other headache syndromes. The most common subtypes of other headache syndromes were “tension-type headache” (G44.2) and “other specified headache syndromes” (G44.8), representing 40.8% (2,385/5,851) and 38.1% (2,230/5,851) of all patients with other headache syndromes, respectively. Data on headache frequency were available for 23.6% (1,381/5,851) of patients, with a median frequency of 8 days per month (range: 0.1–30 days).

### Headache triggers

Among the entire population (FAS), information on headache attack triggers was available for 28.3% (3,127/11,023) of patients. In our study, the most frequently reported migraine attack triggers by patients were menstruation (*n* = 731), movement or intense physical activity (*n* = 660), stress (*n* = 515), skipping meals (*n* = 271), wind or barometric changes (*n* = 210), sleep pattern changes including reduced or excessive sleep (*n* = 302), alcohol consumption (*n* = 140), dehydration (*n* = 55), fatigue (*n* = 29), and exposure to odors, such as perfume (*n* = 24). These findings highlight the diverse range of lifestyle and environmental factors perceived by patients as potential precipitants of migraine attacks.

### Symptoms reported by migraine patients

Headache was the most frequently documented symptom during migraine attacks and was recorded in 8,881 patients. Among accompanying symptoms, nausea was the most commonly reported feature, affecting 4,447 patients (50.1%), followed by photophobia (*n* = 2,327, 26.2%), phonophobia (*n* = 1,694, 19.1%), vomiting (*n* = 1,428, 16.1%), and dizziness (*n* = 1,415, 15.9%). Sensory and neurological symptoms were also frequently observed, including numbness (9.8%), weakness (7.1%), paresthesia (6.5%), visual disturbances such as impaired or blurred vision (6.9% combined), and sleep-related complaints including insomnia and sleep disturbance (8.8% combined). Less commonly reported symptoms included osmophobia, anxiety, tremor, aura, impaired speech, cognitive complaints, and hearing-related symptoms. These findings highlight the broad clinical spectrum of migraine manifestations encountered in routine clinical practice. The full distribution of reported symptoms is presented in Supplementary material 3.

### Concomitant disorders

Among the 11,023 patients included in the Full Analysis Set (FAS), the most frequently recorded concomitant conditions were headache (R51; *n* = 1,606, 14.6%) and tension-type headache (G44.2; *n* = 1,115, 10.1%). Other commonly documented conditions included dizziness and giddiness (R42; *n* = 669, 6.1%), hypertensive heart disease (I11; *n* = 665, 6.0%), vascular headache not elsewhere classified (G44.1; *n* = 453, 4.1%), vitamin D deficiency (E55.9; *n* = 406, 3.7%), essential hypertension (I10; *n* = 384, 3.5%), nausea and vomiting (R11; *n* = 364, 3.3%), and other specified headache syndromes (G44.8; *n* = 379, 3.4%).

Neurological and musculoskeletal disorders such as cervical root disorders (G54.2), cervicalgia (M54.2), carpal tunnel syndrome (G56.0), and polyneuropathy (G63.8) were also frequently recorded. Generalized anxiety disorder (F41.1) was documented in 200 patients (1.8%). These findings highlight the broad spectrum of neurological, cardiovascular, metabolic, and psychiatric conditions encountered in routine migraine care and further underscore the clinical complexity and diagnostic overlap frequently observed in real-world migraine populations. The most frequently recorded concomitant conditions are presented in Table 3.

**Table 3:** Summary of the prescribed therapy by type of treatment regimen, pharmacological subgroup and the most common (≥5% of patients) International Nonproprietary Names (full analysis set).

Treatment regimen pharmacological subgroup (INN)	Verified migraine (*N* = 5,172), *n* (%)	Other headache syndromes (*N* = 5,851), *n* (%)	Total (*N* = 11,023), *n* (%)
Preventive treatment only	1,154 (22.3%)	2,774 (47.4%)	3,928 (35.6%)
Acute treatment only	1877 (36.3%)	1851 (31.6%)	3,728 (33.8%)
Both preventive and acute treatment	2,140 (41.4%)	1,217 (20.8%)	3,357 (30.5%)
Unclassifiable	1 (0.02%)	9 (0.15%)	10 (0.09%)
Attack treatment			
*Pain relievers*	3,337 (64.5%)	2,652 (45.3%)	5,989 (54.3%)
Dexketoprofen	1,044 (20.2%)	853 (14.6%)	1897 (17.2%)
Paracetamol (acetaminophen)	918 (17.7%)	693 (11.8%)	1,611 (14.6%)
Acetylsalicylic acid	486 (9.4%)	379 (6.5%)	865 (7.8%)
Paracetamol (acetaminophen) combinations	416 (8.0%)	348 (5.9%)	764 (6.9%)
*Triptans/Ditans*	1,435 (27.7%)	1,142 (19.5%)	2,577 (23.4%)
Eletriptan	766 (14.8%)	588 (10.0%)	1,354 (12.3%)
Rizatriptan	482 (9.3%)	381 (6.5%)	863 (7.8%)
Frovatriptan	300 (5.8%)	255 (4.4%)	555 (5.0%)
*Anti-emetic drugs*	376 (7.3%)	293 (5.0%)	669 (6.1%)
*Ergot alkaloids*	198 (3.8%)	155 (2.6%)	349 (3.2%)
*Neuroleptics*	40 (0.1%)	8 (0.1%)	12 (0.1%)
Preventive treatment			
*Antidepressants*	1,623 (31.4%)	1,293 (22.1%)	2,916 (26.5%)
Amitriptyline	772 (14.9%)	630 (10.8%)	1,402 (12.7%)
Duloxetine	415 (8.0%)	331 (5.7%)	746 (6.8%)
Venlafaxine	368 (7.1%)	316 (5.4%)	684 (6.2%)
*Beta blockers*	1,005 (19.4%)	787 (13.5%)	1792 (16.3%)
Propranolol	684 (13.2%)	538 (9.2%)	1,222 (11.1%)
*Serotonin antagonists*	286 (5.5%)	241 (4.1%)	527 (4.8%)
*Anti-epileptics*	514 (9.9%)	408 (7.0%)	922 (8.4%)
Topiramate	317 (6.1%)	234 (4.0%)	551 (5.0%)
*Calcium channel blockers*	447 (8.6%)	386 (6.6%)	833 (7.6%)
Flunarizine	402 (7.8%)	346 (5.9%)	748 (6.8%)
*Botulinum toxin*	534 (10.3%)	473 (8.1%)	1,007 (9.1%)
*CGRP monoclonal antibodies*	174 (3.4%)	136 (2.3%)	310 (2.8%)

N: the number of patients in the All-Enrolled Set; n: the number of patients with at least one medication within a specific category. Percentages are calculated as (100 × n/N). Medications are coded by WHO DD version WHO Drug B3.

### Radiological examination

Our findings indicate a high utilization of radiological imaging, with cranial magnetic resonance imaging (MRI) being the most frequently performed modality. Among the 7,219 patients assessed, 4,244 (58.7%) underwent at least one MRI scan, 1,766 (24.4%) had two scans, and 586 (8.1%) had three or more. Head computed tomography (CT) was also frequently used; of the 1,679 patients who underwent CT, 1,284 (76.4%) had one scan, 230 (13.7%) had two scans, and 67 (4.0%) had three or more. These findings suggest a substantial reliance on neuroimaging in the diagnostic evaluation of patients with migraine or suspected migraine, potentially reflecting clinical uncertainty or efforts to exclude secondary causes.

### Patient routing

The majority of patients in both migraine and other headache syndrome groups had outpatient visits as their primary form of medical care. Specifically, 75.4% (3,899/5,172) of migraine patients and 84.2% (4,926/5,851) of patients with other headache syndromes had only outpatient visits. Emergency medical services were utilized by 18.4% (950/5,172) of migraine patients and 8.2% (478/5,851) of patients with other headache syndromes.

### Treatment patterns

The data on prescribed therapies are summarized in Table 4, categorized by treatment regimen type, pharmacological subgroup, and the most common (≥5% of patients) INNs used.

**Table 4:** Most frequently recorded concomitant conditions in the full analysis set (*N* = 11,023).

ICD-10 code	Concomitant condition	*n*
R51	Headache	1,606
G44.2	Tension-type headache	1,115
R42	Dizziness and giddiness	669
I11	Hypertensive heart disease	665
G44.1	Vascular headache, not elsewhere classified	453
E55.9	Vitamin D deficiency, unspecified	406
I10	Essential (primary) hypertension	384
G44.8	Other specified headache syndromes	379
R11	Nausea and vomiting	364
G46.8	Other vascular syndromes of brain in cerebrovascular diseases	340
G54.2	Cervical root disorders, not elsewhere classified	274
R55	Syncope and collapse	270
E61.2	Magnesium deficiency	259
G56.0	Carpal tunnel syndrome	253
E55	Vitamin D deficiency	250
M54.2	Cervicalgia	231
G43.9	Migraine, unspecified	210
F41.1	Generalized anxiety disorder	200
I51	Complications and ill-defined descriptions of heart disease	199
G63.8	Polyneuropathy in other diseases classified elsewhere	193
D51.9	Vitamin B12 deficiency anemia, unspecified	188

N: Number of subjects, ICD: International Classification of Diseases. Multiple concomitant diagnoses could be recorded for the same patient; therefore, the total number of diagnoses exceeds the number of patients.

For patients with a verified migraine diagnosis, 22.3% received preventive treatment exclusively, 36.3% received only acute treatment, and 41.4% received both preventive and acute treatments. Of the patients receiving preventive treatment, 73% were prescribed a single preventive drug (one INN), 20% received two preventive drugs, and 7% received three or more.

The most common pharmacological subgroups prescribed were painkillers (64.5% of patients, with dexketoprofen being the most common), and triptans/ditan (27.7%, with eletriptan as the most common). The most commonly used preventive treatments for patients with migraine are antidepressants (31.4%, with amitriptyline as the most common), and beta blockers (13.2%, propranolol). Additionally, onabotulinumtoxinA injections were prescribed to 10.3% of patients, and CGRP monoclonal antibodies (galcanezumab and erenumab) were prescribed to 3.4% of patients.

In the subgroup of patients with other headache syndromes, 47.4% received preventive treatment only, 31.6% received acute treatment only, and 20.8% received both types of treatment. The most prescribed pharmacological subgroups in this group were painkillers (45.3% of patients), antidepressants (22.1%), and triptans/ditas (19.5%). OnabotulinumtoksinA injections and CGRP monoclonal antibodies were prescribed in 8.1 and 2.3% of cases, respectively.

## Discussion

This large-scale, AI-assisted real-world study provides one of the most comprehensive evaluations of migraine diagnosis and treatment patterns in Türkiye, based on EHRs from over 21,000 patients across 23 private tertiary hospitals and outpatient clinics. Of these, 11,023 patients had documented prescriptions, offering a robust dataset to explore diagnostic accuracy, treatment distribution, and healthcare resource utilization.

### Artificial intelligence for enhanced migraine detection and characterization

By leveraging a validated AI framework, the TMRS was able to extract structured insights from unstructured EHRs, facilitating the identification of patients with verified or suspected migraine. Notably, only 46.9% of patients were formally coded with an ICD-10 G43 diagnosis, despite exhibiting clinical features consistent with migraine. The remaining 53.1% were labeled under alternative headache codes, yet many received migraine-specific treatments, including triptans (19.5%) and botulinum toxin (8.1%). This suggests that, in our cohort, the actual number of patients followed with a migraine diagnosis may be higher than indicated by formal coding. Moreover, the most concomitant code with migraine was headache (R51) in this study and key migraine symptoms such as nausea, photophobia, and phonophobia were underreported compared to structured survey studies like AMPP, likely reflecting limitations in routine clinical documentation (12). The AI-enhanced data extraction process enabled recovery of nuanced clinical information often buried in free-text entries. This approach is supported by prior studies highlighting AI’s superiority in symptom capture, with recall rates exceeding 90% for core migraine features when NLP is applied (8, 13). Riskin et al. aimed to evaluate the use of AI to identify patients with migraine and related symptoms or comorbidities from unstructured clinical data in EHRs. Using NLP, the authors successfully extracted migraine-related information from large-scale EHRs, demonstrating that AI can enhance the detection and characterization of migraine in real-world clinical settings (8). These tools hold promise for standardizing symptom documentation, improving diagnostic coding, and supporting clinical decision-making across diverse practice settings (8, 11, 14–17).

Recent literature has further underscored AI’s potential in headache classification. Studies employing ML models based on patient questionnaires and clinical text have shown high accuracy in distinguishing migraine from other primary headache disorders (18, 19). These models can support non-headache specialists by improving referral quality and reducing diagnostic delays. As the TMRS study illustrates, integrating AI into real-world clinical workflows can bridge gaps between symptoms and diagnosis, expand access to evidence-based care, and enable more targeted therapeutic strategies thereby, paving the way to illustrate heterogenous underpinnings of the disease. Furthermore, this important expansion may provide an opportunity to identify a robust biomarker.

### Demographic findings and clinical features

As in previous epidemiological studies, the majority of patients in our cohort were female (73%) and under the age of 50 (76%), with a median age of 39 years. These findings are consistent with the well-established higher prevalence of migraine among women of reproductive age, likely due to hormonal fluctuations and genetic predisposition (2, 5). In our study, the mean duration of migraine attacks was also longer in women (57.4 h vs. 38.1 h in men), reflecting well-documented sex differences in migraine severity, and symptom burden. These sex-related differences have also been substantiated by multiple studies. For example, a large prospective cohort from the Leiden Headache Clinic reported median migraine durations of 32.1 h in men, compared to 36.7 h in women for non-perimenstrual attacks and 44.4 h for perimenstrual attacks (20). Similarly, a nationwide, home-based study conducted in Türkiye found that women not only experienced longer attacks but also reported more associated symptoms, reinforcing the notion of a greater disease burden in female migraineurs (21).

### Triggers, symptoms, and concomitant conditions

In our study, trigger data were available for 28.3% of patients. Among these, the most frequently reported triggers were menstruation, intense physical activity, and stress. Notably, menstrual migraine was particularly common, consistent with prior literature highlighting its higher disability burden and relative refractoriness to standard acute treatments. The American Migraine Prevalence and Prevention (AMPP) Study similarly identified hormonal changes as a dominant trigger among reproductive-age women, associating it with more frequent attacks and greater functional impairment (22).

Symptoms reported by our patients closely align with the core diagnostic features of migraine in the International Classification of Headache Disorders-3 (ICHD) (23). Although the prevalence of associated symptoms most notably nausea (44.7%), photophobia (21.1%), and phonophobia (13.0%) appears slightly lower than those reported in AMPP (where photophobia and phonophobia were reported by over 50% of patients), this may show differences in documentation quality between structured survey data and routine clinical records, which may reflect documentation practices within EHRs or underreporting due to the retrospective nature of the data (12).

In our cohort, headache (R51) and tension-type headache (TTH; G44.2) were among the most frequently co-coded diagnoses. These patterns may reflect true clinical comorbidities, but they could also indicate diagnostic uncertainty or overlapping symptomatology. Unlike findings from large-scale epidemiological studies, psychiatric comorbidities such as anxiety and depression were not commonly recorded in our EHR-based dataset. This underrepresentation may be attributable to physicians’ reluctance to code psychiatric disorders, particularly in the context of insurance coverage limitations. Given that psychiatric comorbidities are well established as highly prevalent in migraine, this discrepancy underscores a potential limitation of structured EHR documentation in fully capturing the spectrum of disease burden. One particularly noteworthy finding of our study was that the third most common concomitant diagnosis was “dizziness and giddiness” (R42*). This real-world observation highlights the frequency with which patients with migraine report vertigo and dizziness in routine clinical practice, underscoring the importance of assessing vestibular symptoms in this population.

These findings reinforce the need for standardized symptom documentation and greater awareness of the full clinical spectrum of migraine, particularly in real-world clinical settings where underreporting and misclassification may be common. Enhanced use of AI-based tools may improve capture of associated symptoms and comorbidities from unstructured clinical notes, facilitating more comprehensive patient profiling and targeted treatment strategies (8).

### Rationale and concerns around radiological imaging

Despite clinical guidelines discouraging routine neuroimaging in patients with typical migraine presentations without red flags, radiological imaging remains frequently overused (24). In our study, many verified migraine patients underwent at least one cranial MRI or CT; however, a considerable number of patients received repeated scans, with over 2,649 individuals undergoing two or more imaging procedures, which may suggest potential overutilization. This pattern mirrors findings from the Turkish arm of the *My Migraine Voice* survey, where 80% of respondents reported having had brain imaging, with an average of 2.3 scans per patient (25). Overuse is also evident among clinicians themselves: a national Turkish survey revealed that 47.4% of neurologists with migraine had undergone MRI for their own headaches, while a U. S. study by Evans et al. found that nearly 40% of neurologists agreed with ordering imaging for themselves or their family members with headache symptoms even though they acknowledged its limited diagnostic value (26, 27). Collectively, these findings reflect a persistent tendency to overuse imaging, even among medical professionals, likely driven by medicolegal concerns, patient anxiety, and the pursuit of diagnostic reassurance. Enhanced guideline dissemination and educational efforts are needed to curb unnecessary imaging and align practice with evidence-based care.

### Use of emergency services

In our study, 18.4% of patients with verified migraine utilized emergency medical services, indicating possible unmet clinical needs, inadequate access to preventive care, or poor outpatient management. This rate is broadly consistent with international observations. For example, in a large U. S. population-based study, nearly 20–25% of migraine patients reported at least one emergency department (ED) visit annually, often driven by severe, uncontrolled attacks and limited access to specialty care (28). Similarly, European data report ED usage rates ranging from 12 to 30%, particularly in settings lacking integrated headache care pathways (29). Supporting this trend, a recent study from a London teaching hospital found that migraine accounted for a substantial proportion of acute neurology referrals and ED attendances, further emphasizing the strain migraine imposes on unscheduled care services (30).

In Türkiye, the observed ED usage rate may reflect both clinical severity and a tendency among patients to bypass outpatient follow-up in favor of immediate symptomatic relief. It may also be influenced by healthcare system structure, where emergency services are widely accessible and often used as an entry point for care. Collectively, these findings suggest a pressing need for better migraine education, stronger primary care engagement, and improved access to guideline-based outpatient treatment to reduce reliance on emergency services.

### Clinical burden and treatment

The median of 8 monthly headache days (MHDs) observed in our cohort surpasses the guideline-recommended threshold (≥4 MHDs) for initiating preventive therapy (31–33). Historical data from Türkiye indicated that, in 2012, only 4.9% of migraine patients received preventive therapy. In contrast, our recently published study showed that 36.8% of patients with definite migraine and ≥4 MHDs were prescribed preventive treatment (34). In the present cohort, 63.7% of patients were on preventives—a proportion exceeding global averages. This relatively high rate may reflect both the inclusion of patients with other headache syndromes who were also treated preventively and improved access to specialist care within private tertiary centers.

Furthermore, CGRP monoclonal antibodies were prescribed to only 3.4% of verified migraine patients in our cohort, closely mirroring the 5.1% reported in the U. S.-based OVERCOME study (35). In both settings, uptake remained limited in the early post-approval period, likely due to reimbursement restrictions, limited physician familiarity, and concerns about long-term safety and cost-effectiveness.

In our cohort, only 10.3% of patients with verified migraine received onabotulinumtoxinA, indicating relatively limited use despite guideline endorsement for chronic migraine (CM). This rate appears modest when compared to findings from a large U. S.-based retrospective claims analysis by Hepp et al. (36), which demonstrated that onabotulinumtoxinA was associated with significantly lower headache-related healthcare resource utilization than oral prophylactic agents in CM patients (37). These findings highlight the potential clinical and economic advantages of botulinum toxin in appropriately selected patients, underscoring the need to optimize its use in routine migraine care.

In our study, antidepressants were the most frequently prescribed preventive agents (31.4%), surpassing beta-blockers (19.4%) and anti-epileptics (9.9%). This pattern differs from the Turkish subset of the *My Migraine Voice* survey, where beta-blockers (41%) and anti-epileptics (31%) were more commonly used (25). The predominance of antidepressants in our cohort may be partially explained by prescribing behaviors among neurologists themselves. A recent national survey showed that Turkish neurologists and neurology residents often self-manage their own migraine using Selective Serotonin Reuptake Inhibitors, Selective Serotonin and Norepinephrine Reuptake Inhibitors, and beta-blockers even though these agents are not prioritized in major guidelines (26). These choices may reflect perceived tolerability or the high burden of psychiatric comorbidities but also highlight the persistent gap between real-world practice and evidence-based recommendations, emphasizing the need for broader access to mechanism-specific preventive therapies (38). In the present study, while access to outpatient neurology services was high, the underuse of migraine specific agents in a substantial minority of patients suggests ongoing educational and systemic gaps that require targeted interventions.

### Strengths and limitations

This study has several important strengths. It represents one of the first large-scale, AI-assisted analyses of migraine care in Türkiye using electronic health records from multiple tertiary care centers. The multicenter, real-world design enabled the evaluation of routine clinical practice across a large patient population without the selection biases commonly associated with prospective recruitment. Furthermore, the integration of diagnostic codes, prescription data, healthcare utilization metrics, and information extracted from unstructured clinical notes provided a comprehensive overview of migraine management in daily clinical practice.

Nevertheless, several limitations should be acknowledged. First, as with all retrospective EHR-based studies, the accuracy and completeness of the findings depend on the quality of routine clinical documentation. Documentation errors, coding inaccuracies, missing data, and variability in physician recording practices may have influenced the results. Although the AI framework enabled extraction of information from unstructured text, it could only identify information that was present in the source records. Consequently, certain clinical characteristics may have been underrepresented because they were not consistently documented during routine care.

This limitation is particularly relevant for headache triggers. Trigger information was available for only 28.3% of patients, reflecting incomplete documentation within the EHR rather than the true prevalence of migraine triggers. Since trigger-related information is not routinely recorded during every clinical encounter, the frequencies reported in this study likely underestimate the actual burden and diversity of migraine triggers in the study population.

Second, headache classification relied primarily on ICD-10 diagnostic codes and physician documentation. While this approach reflects real-world clinical practice, it may not fully capture the diagnostic nuances of the International Classification of Headache Disorders, 3rd edition (ICHD-3). Similarly, prescription records indicate prescribing behavior but do not confirm medication dispensing, adherence, persistence, or treatment effectiveness.

Third, the study lacked detailed longitudinal treatment data, including dose titration, treatment switching, discontinuation, and long-term outcomes. Consequently, treatment trajectories and comparative effectiveness of different therapeutic strategies could not be assessed. In addition, healthcare services received outside the Acıbadem Health Group network were not captured and may have resulted in incomplete characterization of patients’ overall care pathways.

Another important limitation relates to the study setting. The data were obtained exclusively from a large private tertiary healthcare network. Information regarding patients’ educational attainment, income level, and socioeconomic status was not available in the EHR system and therefore could not be analyzed. However, patients seeking care in private hospitals may differ from the general Turkish population in terms of healthcare access, socioeconomic characteristics, health-seeking behavior, and treatment opportunities. These factors may have influenced diagnostic practices, healthcare utilization, imaging rates, and prescribing patterns. Therefore, the findings may not be fully generalizable to public-sector institutions, rural healthcare settings, or underserved populations.

Finally, the relatively low utilization of newer migraine-specific therapies, such as CGRP monoclonal antibodies, likely reflects reimbursement restrictions and access barriers during the study period rather than the absence of clinical need.

Despite these limitations, the study provides valuable real-world insights into migraine diagnosis, treatment patterns, and healthcare utilization in Türkiye, while highlighting the potential of AI-assisted EHR analyses to generate clinically meaningful evidence from large-scale routine healthcare data.

### Implications and future directions

This study demonstrates how AI can be effectively used to extract real-world insights from large-scale EHR datasets. Importantly, the presence of migraine-specific prescriptions in patients lacking a formal diagnosis suggests that AI models could be trained to flag likely migraine cases, aiding clinicians in timely identification, documentation, and the initiation of an effective treatment. Future work should focus on linking these prescribing behaviors to treatment outcomes, adherence, and patient-reported quality of life.

## Conclusion

This study illustrates the utility of combining structured EHR data with AI to enhance the classification and clinical understanding of primary headache disorders. By capturing real-world treatment patterns across a large tertiary care network, it provides a unique perspective on the current state of migraine management in Türkiye. The findings emphasize not only the clinical gaps and opportunities for improvement but also the value of leveraging health data to inform more data-driven, equitable, and scalable care models. Moving forward, integrating AI into health systems may offer a practical path to optimize migraine care and support proactive, evidence-based decision-making.

## Data Availability

The raw data supporting the conclusions of this article will be made available by the authors, without undue reservation.
