# Supplementary material for: Real-world evidence on migraine treatment in routine clinical practice: insights from the Türkiye migraine registry study using artificial intelligence algorithms
Source: Front Neurol. 2026 Aug 17;17:1848551. doi: 10.3389/fneur.2026.1848551 (PMC13526660; doi:10.3389/fneur.2026.1848551)
Supplement: Supplementary file 1 [file Data_Sheet_1.pdf]

Acibadem Migraine - Traverse Health CDM
Business rules Specification

Introduction

The purpose of this document is to describe the ETL mapping of the proprietary data from Acibadem (dataset 'Migraine') into the Traverse Health Common Data Model.

The Acibadem source data contains 2 files: for each hospital. The dates of events in the data range from Jan 2021 through October 2023. The number of patients in source data meeting the full inclusion criteria is 11023.

Inclusion criterias for the patients were:
1. Patients with G43 and G44 ICD-10 codes and their subtypes
2. Age of a patient at the first visit in observation period >=18
3. Prescription of any medication for acute or preventive treatment of migraine

Traverse Health Common Data Model consists of 7 tables, such as Patient, Visit, Diagnosis, Measurement, Observation, Therapy, Procedure.

Data stored in different Cdm tables can be combined using foreign keys (patient_id and visit_id). Symbol * near a Cdm column name listed in Business rules section means that column is foreign key (single or part of a composite key).

Here are specific data types that are used in Traverse Health data model:

Datatype	Data type description
string	string can contain alpha, numeric, special symbols, and/or white space.
integer	numeric values with no decimals.<br>values may contain negative or positive signs in the first character of the field.<br>negative integer s should start with "-". Positive integer s do not need a sign.
decimal	numeric values that may contain a decimal point.<br>As with integer s, decimal values may contain negative or positive signs.
date	expressed in the format: YYYY-MM-DD where:<br>YYYY = 4-digit year MM = month (1-12) DD = day of month
boolean	Y = yes N = no

Business Rules Summary

1. All values transferred from source dataset to Cdm tables were standardized, regardless of whether they were obtained in the results of ML extraction from the free text columns or were contained in the corresponding columns in the data source. Standardization is unification and harmonization of source parameter names and their values:

- ICD-10 codes were used to standardize concomitant diagnoses.

- ATC codes were used to standardize drug names.

- To standardize surgeries, Surgery_Classes were used.

- To standardize all other values, standard naming words were defined. The mapping between the values as they are stored in the source data and their standard names in two languages (Turkish and English) is stored as separate tables in the database.

2. If parameters were extracted from free text columns additional validation was done, including removing duplicates and checking whether the information refers to the date of the visit.

3. Mapping between target and source tables and columns is provided in the table below, where:

- id – means identifier generated during ETL
- calculated – means that there is no such column in source data and a value was calculated according to business rules
- vocabulary – means that a value was taken from the medical vocabulary
- '-' -means that there is no information in source data, and it is not possible to calculate it within Cdm

Target table	Target column	Source column
patient	patient_id*	id
	date_of_birth	date_of_birth
	gender	gender
	ethnic_group	-
	death_date	death_date
	cause_of_death	-
visit	visit_id*	id
	patient_id*	patient_id
	visit_start_date	diagosis_date
	visit_end_date	-
	visit_type	calculated
	department	department
	doctor_speciality	doctor_speciality
	care_site_id	hospital_branch

diagnosis	diagnosis_id	id
	patient_id*	patient_id
	visit_id*	visit_id
	diagnosis_date	diagosis_date
	diagonosis_type	calculated
	diagnosis_code	all_diagnosis_codes_for_this_visit,<br>hypertension,<br>cardiovascular_desease,<br>diabetes,<br>blood_disease,<br>other_chronic_deseases
	diagnosis_name	vocabulary
	diagnosis_status	diagnosis_status
measurement	measurement_id	-
	patient_id*	-
	visit_id*	-
	measurement_date	-
	measurement_name	-
	measurement_parameter	-
	text_result	-
	numeric_result_operator	-
	numeric_result	-
	unit_of_measure	-
	range_operator	-
	high_range	-
	low_range	-
observation	observation_id	id
	patient_id*	patient_id
	visit_id*	visit_id
	observation_date	diagosis_date

	observation_type	calculated
	observation_name	height,<br>weight,<br>blood_group,<br>disability,<br>alcohol,<br>smoking,<br>allergy,<br>complaints,<br>patient_history,<br>inspection_note,<br>control_note,<br>treatment_note,<br>medical_note
	value_as_string	
	value_as_number	
	unit_of_numeric_value	calculated
therapy	therapy_id	id
	patient_id*	patient_id
	visit_id*	visit_id
	drug_name	calculated
	drug_code	calculated
	therapy_type	calculated
	start_date	diagnosis_date for outpatient visits<br>drug_order_date for inpatient visits
	end_date	
	days_supply	
	quantity	
	dose	
	dose_unit	
	route	
procedure	procedure_id	id
	patient_id*	patient_id
	visit_id*	visit_id
	procedure_name	surgical_history
	procedure_date	diagnosis_date

Data Mapping

PATIENT

Overview

This table is to be populated with basic patient demographic information such as date of birth and gender for each patient and there has to be one and only one record per unique patient identifier.

Business rules

1. Patients whose minimal age at the beginning of observation period was less than 18 were removed from the source data set.

2. Patients who did not receive targeted therapy during the observation period were removed from the dataset.

Field name	Datatype	Is required	Guide
patient_id*	integer	Y	unique patient identifier generated during ETL
date_of_birth	date	Y	src. date_of_birth
gender	string	Y	when src.gender = 'E' then 'Male'<br>when src.gender = 'K' then 'Female'<br>else 'Other'
ethnic_group	string		NULL
death_date	date		src.death_date
cause_of_death	string		NULL

VISITS

Overview

There has to be one and only one record per patient encounter, which may represent a visit to an emergency room, a hospital admission, an outpatient visit, etc. Each diagnosis, procedure, lab result record, etc., should be associated with a valid encounter record.

Business rules

1. Most patients have only 1 visit during the observation period.

2. There is a mix of visit types and department names in the source 'Visit type' column. If visit type in source data is Inpatient ('Yatış') then we assigned 'Inpatient' visit type in cdm table, if visit type in source data is Emergency Department then we assigned 'Emergency' visit type in cdm table, for those values where

department name is other then Inpatient and Emergency Department we assigned 'Outpatient' visit type in cdm table. So, there are 3 visit types in cdm Visit table: Outpatient, Inpatient, Emergency.

3. There is no distinction between diagnosis date and visit date in the source data. Visit dates were set to diagnosis dates in cdm tables.

Field name	Datatype	Is required	Guide
visit_id*	integer	Y	the unique key given to each record in this table<br><br>visits with the same visit_id and different visit_date are defined as one visit if all other information is the same - in this case, the maximum date of the visit is selected.
patient_id*	integer	Y	cdm.patient_id
visit_start_date	date	Y	src.diagnosis_date
visit_end_date	date		NULL
visit_type	string	Y	when t.visit_type = 'Yatış' and ss.standard_name_english <> 'Emergency Department' then 'Inpatient'<br><br>when ss.standard_name_english = 'Emergency Department' then 'Emergency'<br><br>else 'Outpatient'
department	string		src. visit_type<br>standardized english value
doctor_speciality	string		NULL
care_site_id	integer	Y	the unique key given to each unique src.hospital_branch

DIAGNOSIS

Overview

This table contains records of Events of a Person suggesting the presence of a disease or medical condition stated as a diagnosis which is either observed by a Provider or reported by the patient.

A sign, a complaint or a symptom observed by a Provider or reported by the patient are recorded in the cdm.Observation table since it is not possible to get appropriate ICD-10 codes for them and therefore they cannot be part of cdm.Diagnosis table.

Business rules

1. The information about diagnosis is contained in many columns of source table such as: all_diagnosis_codes_for_this_visit, hypertension, cardiovascular_desease, diabetes, blood_disease, other_chronic_deseases.
   - The column all_diagnosis_codes_for_this_visit contains one or more icd10 codes separated by a comma delimiter. These codes can be both main and concomitant (or preliminary) diagnosis. In order to define main diagnosis at the visit level, the following bussines rule was applied:
     - ICD10 code is G43(.x) and department name is one of neurological type of departments (the list of departments is provided in the table below); all other ICD10 codes for this visit are concomitant diagnosis.
     - ICD10 code is G44(.x) and there is no G43(.x) at the visit and department name is one of neurological type of departments (the list of departments is provided in the table below); all other ICD10 codes for this visit are concomitant diagnosis.
     - In rare cases, when there were multiple different ICD10 codes G43 during one visit, the main diagnosis was selected based on a more prioritized principle (for example, migraine with aura takes priority over migraine unspecified); if it was not possible to select a more prioritized code (for example, if both migraine with aura and migraine without aura were present simultaneously), then a common code G43 was assigned for such cases. Similarly, the selection of one main code between subtypes G44 followed the same principle.
   - The columns hypertension, cardiovascular_desease, diabetes, blood_disease contain 'Yes' value for patients who have the corresponding diseases. These are concomitant chronic diseases recorded in the appropriate section by the Provider during the visit.
   - The column other_chronic_deseases contains other chronic diseases as a free text. Appropriate icd10 code was assigned for each separate disease. These are also concomitant diseases.
2. Diagnosis status (Preliminary, Final) is not always precise in source data since doctors often forget to change the status. It is not possible to apply any business rules to make the information more accurate (mainly because most patients have only one visit during the observation period), therefore, it remains untouched, although not always accurate.
3. Diagnosis date is equal to visit date in source data. Diagnosis date for concomitant diseases is absent in source data, it was set equal to visit date in cdm table.

Field name	Datatype	Is required	Guide
diagnosis_id	integer	Y	the unique key given to each record in this table
patient_id*	integer	Y	cdm.patient_id`
visit_id*	integer	Y	cdm.visit_id
diagnosis_date	date	Y	src. diagnosis_date<br><br>convert to date format
diagonosis_type	string	Y	1) for the column src.all_diagnosis_codes_for_this_visit<br><br>set diagnosis type as Main if<br><br>1. icd10 code is G43(.x) or icd10 code is G44(.x) (and

			there is no G43(.x) for the visit)<br>2. department name is one of ('Neurology','Neurosurgery','Emergency Department','Migraine Treatment','Pediatric Neurology','Psychiatry','Headache Clinic', 'Pain Management')<br><br>set diagnosis type as Concomitant for the rest<br><br>2) for diagnosis derived from other columns set diagnosis type as Chronic
diagnosis_code	string		source 1) take distinct icd-10 codes from the column src.all_diagnosis_codes_for_this_visit<br><br>source 2) if value in the columns below = 'Evet' (=Yes) then create a record in cdm.Diagnosis table with appropriate icd-10 codes :<br>• hypertension (assign diagnosis_code I11)<br>• cardiovascular_desease (assign diagnosis_code I51)<br>• diabetes (assign diagnosis_code E14)<br>• blood_disease (assign diagnosis_code D75.9)<br>• other_chronic_deseases (find appropriate codes in the table in the Appendix section)
diagnosis_name	string	Y	lookup v.concept_name in v.omop_cdm_schema.concept table<br>where vocabulary_id = 'ICD10'<br>and cdm.diagnosis_code = v.concept_code
diagnosis_status	string		src.diagnosis_status:<br><br>when src.diagnosis_status = 'Ön Tanı' then 'Preliminary'<br>when src.diagnosis_status = 'Kesin Tanı' then 'Final'

MEASUREMENTS

Overview

Populate this table with laboratory tests, vital signs, quantitative findings from pathology reports, imaging. Measurements differ from Observations in that they require a standardized test or some other activity to generate a quantitative or qualitative result.

Business rules

We do not populate this table due to absence of information in source data!

Field name	Datatype	Is required	Guide
measurement_id	integer	Y	The unique key given to each record in

			this table
patient_id*	integer	Y	
visit_id*	integer	Y	
measurement_date	date		
measurement_name	string		
measurement_parameter	string		
text_result	string		
numeric_result_operator	string		
numeric_result	decimal		
unit_of_measure	string		
range_operator	string		
high_range	decimal		
low_range	decimal		

OBSERVATIONS

Overview

The OBSERVATIONS table captures clinical facts about a Person obtained in the context of examination, questioning or a procedure. Any data that cannot be represented by any other domains, such as physical examinations (height, weight, BMI), social and lifestyle facts, profession, social status, financial status, medical history, family history, heredity, questionnaires, etc. are recorded here.

Business rules

1. There are many columns in source data where information about observations is stored. In most cases this is a separate column for each observation with corresponding column name (for example in the source column 'Allergy' information about allergy is stored and so on). However, some of observations are recorded in free text columns (such as complaints, patient_history, inspection_note, control_note, treatment_note, medical_note) along with other information about the patient (for example, the medical history column contains a lot of diverse information about the patient in the form of free text).

2. If observations were extracted from free text columns additional validation was done, including removing duplicates and checking the accuracy of the ML extract.

3. Cdm Observation table contains the following observations:

Observation_type	Observation_name	Description

Alcohol/Smoking	Alcohol use	
Alcohol/Smoking	Smoking	
Allergy	Allergen	
Anthropometric indicators	Blood group	
Anthropometric indicators	BMI	
Anthropometric indicators	Height	
Anthropometric indicators	Weight	
Complaints/Symptoms	Complaints, Symptoms	
Complaints/Symptoms	Headache_duration	Duration of continuous headache event
Complaints/Symptoms	Headache_frequency	
Complaints/Symptoms	Headache_intensity	
Complaints/Symptoms	Headache_timespan	Total timespan of headaches from start of headache events to current visit. There should be multiple independent events within this period of time
Complaints/Symptoms	Triggers	
Medical history	Disability	
Neurogical_examination	Neurogical_examination_total_result	
Observation	Number of CT	
Observation	Number of MRI	

4. There is no information about observation dates in the source data, so observation dates were set equal to diagnosis dates (aka visit dates) in Cdm Observation table.

5. Information about the Neurological statuses was obtained by extracting the corresponding parameters and their values from the free text columns. Different parameters within the same neurological examination may have different values (Normal, Abnormal). In order to determine the neurological status for the entire examination, the following rule was applied: if at least one of the parameters has the abnormal result, the status of the entire neurological examination is defined as Abnormal. The status of the entire neurological examination is defined Normal only if all the parameters inside the examination have normal results.

6. The values of the headache timespan, duration and frequency were obtained as a result of free text parsing:

- In some cases, the values in the free text were specified as a range (for example, “2-4 hours”). In this case, the upper limit of the range was taken as the value. Also, if the timespan or duration was mentioned several times in different parts of the same free text or in different columns, but within the same visit, and if the obtained values were different, the maximum value was selected.

- For visits containing “days” statements, firstly if there are “seconds”, “minutes” or “hours” within the same visit than the “days” statement was accepted as “timespan”, secondly if there are “weeks”, “months” or “years” within the same visit than the the “days” statement was accepted as “duration”, finally if the upper range of “days” statement is lower than or equal to “1” than the value accepted as “duration”.

- Headache duration values could be specified as seconds, minutes, hours, or days. To unify the values all duration values in the CDM table have been converted to hours.

- Headache timespan could be specified as days, weeks, months, or years. To unify the values, all timespan values in the CDM table were converted to months.

- To unify headache frequency values, all values in the CDM table were converted to days per month.

Field name	Datatype	Is required	Guide
observation_id	integer	Y	the unique key given to each record in this table
patient_id*	integer	Y	cdm.patient_id
visit_id*	integer	Y	cdm.visit_id
observation_date	date	Y	src.diagnosis_date
observation_type	string	Y	assigned during ETL as names for groups of observations (for example observations names Height, Weight, BMI, Blood group were grouped into one observation type ‘Anthropometric indicators’)
observation_name	string	Y	assigned during ETL<br>1. based on a column name where observation value was derived from (for example a source column where information on smoking is stored got observation name ‘Smoking’)<br>or<br>2. based on the parameter that was extracted from the free text
value_as_string	string		result of an observation if it is in text format
value_as_number	decimal		result of an observation if it is in numeric format
unit_of_numeric_value	string		unit of measures of numeric result of observation (if applicable)

THERAPY

Overview

This table captures records about the exposure to a Drug ingested or otherwise introduced into the body. A Drug is a biochemical substance formulated in such a way that when administered to a Person it will exert a certain biochemical effect on the metabolism. Drugs include prescription and over-the-counter medicines, vaccines, and large-molecule biologic therapies. Radiological devices ingested or applied locally do not count as Drugs.

Business rules

1. Information comes from source columns prescribed_medications, treatment_note, medical_note, inspection_note, control_note, patient_history, regularly_used_medications, complaint. All but prescribed_medications are unstructured, free text columns that require ML natural language processing to extract information from them.
2. Initial parsing of drugs from raw data is done in Python: through a simple custom script for prescribed_medications column, using a ML model for the others.
3. For drug information extracted via ML model, additional validation was performed, including removal of duplicates and verifying accuracy of parsing against original text.
4. The active ingredients and ATC code of the drug are found via scraping the internet resources, mainly ilacabak.com.
    - In case the medication in question does not have an ATC code (e.g., complex vitamin supplements), only active ingredient(s) are listed.
    - When the active ingredient(s) have several matching ATC codes within the same subgroup (e.g., unspecified insulin type), a 3rd/4th level ATC code is used.
    - When the active ingredient(s) have several matching ATC codes that do not share a subgroup (e.g., the drug has different ATC codes depending on administration route) and the type cannot be ascertained, ATC code is left unfilled and the drug is identified only by the active ingredient(s).
5. Active ingredient(s) that could not be identified definitively via scraping the web were filled in manually unless they are very rare (less than 10 total occurrences in the entire dataset).

Field name	Datatype	Is required	Guide
therapy_id	integer	Y	The unique key given to each record in this table
patient_id*	integer	Y	cdm.patient_id
visit_id*	integer	Y	cdm.visit_id
drug_name	string	Y	The active ingredient(s) of the drug
drug_code	string		The ATC code of active ingredient in accordance with the WHO methodology (where can be identified)

therapy_type	string		3 types:<br>• 'current' - prescription and instructions written at the *current* visit, i.e. patient should be taking them starting from this visit (source columns: prescribed_medications, treatment_note)<br>• 'regular' - drugs the patient reports to be taking *habitually prior to* this visit and *likely to continue* using. Can be both concomitant and migraine-related (source column: regularly_used_medications)<br>• 'previous' - drugs the patient reports using *prior to this visit* or those noted by the doctor (source columns: patient_history, inspection_note, control_note, medical_note, complaint)
start_date	date	Y	src.diagnosis_date<br>- visit date. 'current' therapy starts on this date, 'previous' is reported as having taken place before it.
end_date	date		
days_supply	decimal		The intended duration of the prescription, in days
quantity	decimal		
dose	decimal		The dose of the drug given
dose_unit	string		The dose unit of the drug given
route	string		Route of administration, i.e. 'oral'

PROCEDURES

Overview

This table contains records of activities or processes ordered by, or carried out by, a healthcare provider on the patient with a diagnostic or therapeutic purpose.

Business rules

1. All information about the surgeries was taken from the source column ‘surgical_history’. This is a free text column and rule-based text mining algorithms have been applied to extract surgery names from it. To standardize the names of surgeries, official names of the surgery classes from Turkish Health Ministry were used, since in many cases there was not enough detailed information to determine the exact and detailed name of the surgery.

2. There is no information about the date of the procedure (surgery) in the source data. The date of diagnosis (aka the date of the visit) was set as the date of the procedure. Therefore, the date of the procedure in this case should be understood as the date when a record about the procedure was made by Provider in the medical system.

Field name	Datatype	Is required	Guide
procedure_id	integer	Y	the unique key given to each record in this table
patient_id*	integer	Y	cdm.patient_id
visit_id*	integer	Y	cdm.visit_id
procedure_name	string	Y	ml. class_name_english<br><br>(split by comma delimeter if there are multiple surgeries at a visit)
procedure_date	date	Y	src.diagnosis_date

Appendix

Appendix 1. Data dictionary

Data dictionary defines each of the items on which data is collected and provides information on how the required data is sourced and entered in the database. Data items are listed in the order in which they are entered on the database.

Patient data:

Field name	Datatype	Is required	Description	Allowed Values
patient_id*	integer	Y	unique patient identifier generated during ETL	
date_of_birth	date	Y	the date of birth if it is known	DD-MM-YYYY
gender	string	Y	Gender of the patient	Male, Female
ethnic_group	string		NULL	N/A
death_date	date		the date of death if it is known	DD-MM-YYYY
cause_of_death	string		NULL	N/A

Visit data:

Field name	Datatype	Is required	Description	Allowed Values
visit_id*	integer	Y	the unique key given to each record in this table	
patient_id*	integer	Y	unique patient identifier generated during ETL	
visit_start_date	date	Y	Date of the patient's visit	DD-MM-YYYY
visit_end_date	date		NULL	N/A
visit_type	string	Y	service type of admitted patient	'Inpatient', 'Outpatient' or 'Emergency Department'
department	string		Department name	
doctor_speciality	string		NULL	N/A
care_site_id	integer	Y	hospital_branch unique key	1-30

Diagnosis data:

Field name	Datatype	Is required	Description	Allowed Values
diagnosis_id	integer	Y	the unique key given to each record in this table	
patient_id*	integer	Y	Unique identifier for each patient	
visit_id*	integer	Y	Unique identifier for each visit	
diagnosis_date	date	Y	Diagnosis date	DD-MM-YYYY
diagonosis_type	string	Y	Type of diagnosis	Main, Concomitant, Chronic
diagnosis_code	string		ICD-10 diagnosis code	
diagnosis_name	string	Y	ICD-10 diagnosis title	
diagnosis_status	string		Status of the diagnosis	'Preliminary', 'Final'

Observation data:

Field name	Datatype	Is required	Description	Allowed Values
observation_id	integer	Y	the unique key given to each record in this table	
patient_id*	integer	Y	Unique identifier for each patient	
visit_id*	integer	Y	Unique identifier for each visit	
observation_date	date	Y	Date of the performed observation	DD-MM-YYYY
observation_type	string	Y	Groups of observation	'Alcohol/Smoking', 'Allergy', 'Anthropometric indicators', 'Complaints/Symptoms', 'Medical history', 'Neurogical_examination', 'Observation'
observation_name	string	Y	Name of the observation	
value_as_string	string		Text result of an observation	
value_as_number	decimal		Numeric result of an observation	
unit_of_numeric_value	string		Measures unit of numeric result of observation	

Therapy data:

Field name	Datatype	Is required	Description	Allowed Values
therapy_id	integer	Y	The unique key given to each record in this table	
patient_id*	integer	Y	Unique identifier for each patient	
visit_id*	integer	Y	Unique identifier for each visit	
drug_name	string	Y	The active ingredient of the drug	
drug_code	string		ATC code of active ingredient	
therapy_type	string		Therapy relationship to current visit	'current', 'regular', 'previous'
start_date	date	Y	Therapy start date	DD-MM-YYYY
end_date	date			
days_supply	decimal		The intended duration of the prescription, in days	
quantity	decimal			
dose	decimal		The dose of the drug	
dose_unit	string		The dose unit of the drug	
route	string		Route of administration	

Procedure data:

Field name	Datatype	Is required	Description	Allowed Values
procedure_id	integer	Y	the unique key given to each record in this table	
patient_id*	integer	Y	Unique identifier for each patient	
visit_id*	integer	Y	Unique identifier for each visit	
procedure_name	string	Y	Diagnostic or Therapeutic procedure name	
procedure_date	date	Y	Date of the procedure performed	DD-MM-YYYY

Lab data:

Field name	Datatype	Is required	Description	Allowed Values
procedure_id	integer	Y	the unique key given to each record in this table	
patient_id*	integer	Y	Unique identifier for each patient	
visit_id*	integer	Y	Unique identifier for each visit	
procedure_name	string	Y	Diagnostic or Therapeutic procedure name	
procedure_date	date	Y	Date of the procedure performed	DD-MM-YYYY
