# Supplementary material for: Real-world evidence on migraine treatment in routine clinical practice: insights from the Türkiye migraine registry study using artificial intelligence algorithms
Source: Front Neurol. 2026 Aug 17;17:1848551. doi: 10.3389/fneur.2026.1848551 (PMC13526660; doi:10.3389/fneur.2026.1848551)
Supplement: Supplementary file 2 [file Data_Sheet_2.pdf]

STATISTICAL ANALYSIS PLAN

Turkiye Migraine Registry Study

Study No.: TMRS-2023

EFFECTIVE DATE: 08-Feb-24

VERSION/STATUS: Version 1.1

CONFIDENTIALITY STATEMENT

The information contained in this document is privileged and confidential.

Do not copy, circulate or otherwise distribute without written authority from Traverse Health Europe B.V.

SAP APPROVAL

By my signature, I confirm that this SAP has been reviewed by TRAVERSE HEALTH VERİ HİZMETLERİ LİMİTED ŞİRKETİ, and has been approved for use on the TMRS-2023 study.

Alex Loleit, Chief Executive Officer, TRAVERSE HEALTH VERİ HİZMETLERİ LİMİTED ŞİRKETİ

Signature   Date

Table of contents

1. INTRODUCTION 5
2. PROJECT OVERVIEW 6
2.1 Study Objective 6
2.2 Methodology, data source and analyzed period 6
2.3 Study population 7
2.4 Sample size 7
3. ANALYSIS SETS/ POPULATIONS 8
3.1 Full Analysis Set 8
3.2 Subgroup analysis 8
4. STATISTICAL CONSIDERATIONS 10
4.1 General Considerations 10
5. ANALYSES AND SUMMARIES 11
5.1 Analysis of Categorical Variables 11
5.2 Demography and anthropometry 11
5.3 Main disease characteristics 11
5.3.1 Main disease profile 11
5.3.2 Subtypes of verified migraine 11
5.3.3 Subtypes of other headache syndromes 11
5.3.4 Headache attacks 12
5.3.5 Non-pain-associated symptoms 12
5.4 Concomitant diseases 12
5.5 Medication patterns/ medications of interest 12
5.6 Patient routing 13
5.7 Other 13
6. REFERENCES 14

List of Abbreviations and Definitions

Abbreviation	Description
RWE	Real-World Evidence
EHRs	Electronic Health Records
AI	Artificial Intelligence
NLP	Natural Language Processing
AE	Adverse Event
ED	Emergency Department
INNs	International Nonproprietary Names
SD	Standard Deviation
ATC	Anatomical Therapeutic Chemical

1. INTRODUCTION

Migraine is a disabling primary headache disorder that directly affects over 1 billion people across all world regions, cultures, and socioeconomic statuses. Migraine incidence studies performed throughout the world report rates of 2-10/1000 in men and 3-18/1000 in women, whereas the rates for Turkey is much higher due to both the genetic background and the impact of environmental factors, and was reported as 29.8/1000 in women and 19.3/1000 in men. Local Turkish studies shows that migraine lead to limitations in both individual and professional life at each stage, including prodrome, headache and postdrome phases, particularly in a patient group who have ≥4 days with migraine headaches a month and failed to achieve sufficient benefit from the prophylactic therapies they have used who can be classified as to have "severe" disease.

In recent years, the need to support informed decision making in treatment, payer policy, and regulation has led policy makers to broaden efficacy information they seek from randomized placebo-controlled clinical trials with limited generalizability and consider a focus on real-world evidence (RWE) collected in routine clinical practice. The goal of this augmentation is to gain insights into the effectiveness and efficiency of clinical treatments at a population level outside of results derived in a high-resource clinical trial setting studying a stringently defined disease population. RWE is increasingly employed not only to understand real-world outcomes, but also to power studies sufficiently to enable subgroup analytics, comparative effectiveness, and tailored treatment plans.

In disease areas with 'soft' outcomes (i.e., the subjective aspects of a medical condition or its management) such as migraine, extraction and validation of real-world evidence from electronic health records (EHRs) and other routinely collected data can be challenging due to how the data are collected and recorded.

Traverse Health defined and validated a scalable framework model to measure outcomes of migraine treatment by use of artificial intelligence (AI) algorithms within EHR unstructured data. The developed approach includes the deployment of natural language processing (NLP) and machine learning algorithms, both aspects of artificial intelligence.

2. PROJECT OVERVIEW

2.1 Study Objective

To evaluate prevalence of different types of migraine, to assess medication patterns, patient characteristics, and healthcare resource utilisation in routine clinical practice.

2.2 Methodology, data source and analyzed period

This is non-interventional retrospective study based on anonymized patients' medical records.

In this non-interventional study retrospective data of routine clinical practice is collected in the absence of intervention (interventions) and control.

The data sources (integrated electronic medical records of patients) and / or clinical centers included in this project are selected administratively, taking into account the organizational and financial capabilities.

Automated identification of patients who have had an index event during the analyzed period and who meet the selection criteria is performed in the database of integrated medical records of patients.

The total analyzed period (the period for which data is collected for analysis): from 1st January 2021 to 31st October 2023.

The index date/event in this study is the earliest reported case of inpatient or outpatient treatment (referral) of migraine during total analyzed period.

This query/analysis involves a combination of existing structured data and unstructured data, which will be converted to structured form solely by a computer using automated/algorithmic methods, such as natural language processing.

In these data sources, individual patient data are not retrieved or validated, and it is not possible to link (i.e., identify a potential association between) a particular product and medical event for any individual. Thus, the minimum criteria for reporting an adverse event (AE) (i.e., identifiable patient, identifiable reporter, a suspect product, and event) cannot be met.

The data provider for this study is the Acibadem hospitals network in Turkiye. The raw data was obtained from 23 Acibadem hospitals. Both inpatient and outpatient visits are included in the raw data. Subsequent ETL work was carried out to transform raw data into the Traverse Health Common Data Model, which includes: raw data validation, duplicate removal, parsing free text or extracting parameters using ML, transferring data to the Traverse Health Common Data Model while adhering to the requirements of the Common Data Model regarding format and mandatory values, harmonizing and encoding values, handling missing data, testing, and documenting the entire ETL process in a separate document "Acibadem Migraine - Traverse Health CDM Date Rules Specification".

2.3 Study population

Selection criteria

Eligible patients are identified with the following inclusion criteria:

1. Men and women aged 18–100 years.
2. Documented diagnosis of "Migraine" (ICD-10: G43) or other headache syndromes (ICD-10: G44).
3. Prescription of any medication for acute or preventive treatment of migraine*.

*** List of target INNs (Source: [5-7]):**

Preventive treatment

- Monoclonal antibodies against calcitonin gene-related peptide (CGRP) or its receptor (INNs: erenumab, galcanezumab, fremanezumab, eptinezumab)
- Botox injections (INN: botulinum toxin)
- Beta blockers (INNs: propranolol, atenolol, metoprolol, timolol, nadolol, bisoprolol, nebivolol)
- Calcium channel blockers (INNs: verapamil, flunarizine)
- Antidepressants (INNs: amitriptyline, fluoxetine, venlafaxine, duloxetine)
- Anti-epileptics (INNs: valproic acid, topiramate, gabapentin)
-

Acute treatment

- Pain relievers (INNs: acetylsalicylic acid, paracetamol, diclofenac, flurbiprofen, ibuprofen, dexibuprofen, dexketoprofen, ketoprofen, ketorolac, metamizole sodium, naproxen, phenazone, tolfenamic acid, hydromorphine, meperidine, morphine, butorphanol, mefenamic acid, pethidine, fentanyl, oxycodone, tramadol).
- Triptans/Ditans (INNs: almotriptan, eletriptan, frovatriptan, naratriptan, rizatriptan, sumatriptan, zolmitriptan, lasmiditan).
- Combination triptan + NSAID
- Ergot alkaloids (INNs: ergotamine, dihydroergotamine ergotamine).
- Neuroleptics (INNs: chlorpromazine, prochlorperazine, promethazine)
- Anti-emetic drugs (INNs: domperidone, metoclopramide, trimethobenzamide, dimenhydrinate, granisetron, ondansetron, palonosetron).
- diclofenac, thiocolchicoside, chlorzoxazone, propyphenazone, combinations excl. psycholeptics

2.4 Sample size

This study is exploratory and does not involve testing of statistical hypothesis.

It is expected that the dataset will include about 12000 patients. This number of patients is sufficient to carry out the planned data analysis.

3. ANALYSIS SETS/ POPULATIONS

3.1 Full Analysis Set

The main statistical analysis will be performed in the Full Analysis Set (FAS). FAS will include all eligible patients (see inclusion criteria in Section 2.3)

3.2 Subgroup analysis

Patients will be categorized into subgroups based on the following:

- by year of the index event/date:
    - 2021
    - 2022
    - 2023

- by gender:
    - male
    - female

- by age category:
    - 18-24
    - 25-29
    - 30-34
    - 35-39
    - 40-44
    - 45-49
    - 50-54
    - 55-59
    - 60-64
    - 65-70
    - 18-70
    - 70+

- by main disease profile:
    - verified diagnosis of migraine (the presence of the "ICD-10: G43" code)
    - suspected diagnosis of migraine (other headache syndromes - the presence of the "ICD-10: G44" code or "ICD-10: R51" code and absence of the "ICD-10: G43" code)

- by index migraine subtype:
    - migraine without aura (G43.0)
    - migraine with aura (G43.1)
    - other migraine (G43.8)
    - migraine, unspecified (G43.9+G43)
    - complicated migraine (G43.3)
    - status migrainosus (G43.2)

- by index other headache syndrome:
    - other headache syndromes, unspecified (G44)
    - cluster headache syndrome (G44.0)

- vascular headache, not elsewhere classified (G44.1)
- tension-type headache (G44.2)
- chronic post-traumatic headache (G44.3)
- drug-induced headache, not elsewhere classified (G44.4)
- other specified headache syndromes (G44.8)

- by treatment regime:
  - both preventive and acute treatment
  - only acute treatment
  - only preventive treatment

- by study site:
  - separately for each hospital

4. STATISTICAL CONSIDERATIONS

4.1 General Considerations

Categorical variables will be presented as frequencies and proportions in percent. Continuous variables will be presented as mean, standard deviation, median, minimum and maximum, and quartiles (where applicable), with number of observations.

The minimum and maximum values will be displayed to the same decimal precision as the source data, the arithmetic mean, median, quartiles values will be displayed to one more decimal than the source data, and the SD values will be displayed to two more decimals than the source data for the specific variable. Percentages will be rounded to one decimal place.

The maximum number of decimal symbols in the statistical report is four. If some descriptive statistical value has more than four decimal symbols after above mentioned rules application, this value will be rounded to four decimal symbols.

The appropriate precision for derived variables will be determined based on the maximum precision of the data on which the derivations are based, and statistics will be presented in accordance with the above-mentioned rules.

The data and results of the analyses will be presented in the form of tables, figures and listings.

5. ANALYSES AND SUMMARIES

5.1 Analysis of Categorical Variables

Unless otherwise written and applicable, then for all categorical variables will be presented tables with frequency and percentage, frequency histogram, percentage histogram for the Full Analysis Set and by all subgroups listed in Section 3.2.

Also, tables with frequency and percentage, frequency histogram, percentage histogram with divided by combination of each pair of groups will be presented.

5.2 Demography and anthropometry

Descriptive data will be provided for the demography (age, gender) and anthropometry (weight, body mass index and height) for the Full Analysis Set and for all subgroups listed in Section 3.2 The age will be also described as categorical data by categories presented in Section 3.2.

Also, will be presented separated analysis of number and demographic characteristics of patients who have presented to the Emergency Department (ED).

5.3 Main disease characteristics

5.3.1 Main disease profile

- Verified diagnosis of migraine (the presence of the "ICD-10: G43" code)
- Suspected diagnosis of migraine (the presence of other headache syndromes (ICD-10: G44).

Descriptive data will be provided according to Section 5.1.

5.3.2 Subtypes of verified migraine

- migraine without aura (G43.0)
- migraine with aura (G43.1)
- other migraine (G43.8)
- migraine, unspecified (G43.9+G43)
- complicated migraine (G43.3)
- status migrainosus (G43.2)

Descriptive data will be provided according to 5.1.

5.3.3 Subtypes of other headache syndromes

- other headache syndromes, unspecified (G44)
- cluster headache syndrome (G44.0)
- vascular headache, not elsewhere classified (G44.1)
- tension-type headache (G44.2)
- chronic post-traumatic headache (G44.3)
- drug-induced headache, not elsewhere classified (G44.4)
- other specified headache syndromes (G44.8)

Descriptive data will be provided according to Section 5.1.

5.3.4 Headache attacks

The frequency (per month), and duration of headache attacks and numeric pain scale will be analyzed according Section 4.1 by subgroups listed in Section 3.2. Also, will be presented the box plot with age category on abscissa axis and mean ± standard deviation by subgroups-variables and overall, on ordinate axis. Intensity of headache will be analysed according to Section 5.1.

5.3.5 Non-pain-associated symptoms

Descriptive data for the nausea, vomiting, photophobia, phonophobia, aura, and other symptoms will be provided according to Section 5.1 without histograms.

5.4 Concomitant diseases

Descriptive data for the concomitant neurological diseases (epilepsy, stroke, multiple sclerosis etc), mood disorders, suicidal ideation, cardiovascular disorders, obesity, diabetes, other endocrine disorders, bronchial asthma, cancer, immunosuppression, autoimmune diseases, surgeries, etc will be provided according to 5.1. Other diagnoses received by these patients as depression, anxiety, fibromyalgia, asthma, heart disease, vertigo, sleep disorders, BPPV, etc will be analysed separately.

5.5 Medication patterns/ medications of interest

Targeted therapy

The number and percentage of participants receiving targeted medications (by therapy type (preventive or acute)) and other concomitant medication coded by ATC classification system codes and INNs will be analysed according to Section 5.1.

Preventive treatment

- Monoclonal antibodies against calcitonin gene-related peptide (CGRP) or its receptor (INNs: erenumab, galcanezumab, fremanezumab, eptinezumab)
- Botox injections (INN: botulinum toxin)
- Beta blockers (INNs: propranolol, atenolol, metoprolol, timolol, nadolol)
- Calcium channel blockers (INNs: verapamil, flunarizine)
- Antidepressants (INNs: amitriptyline, fluoxetine, venlafaxine, duloxetine)
- Anti-epileptics (INNs: valproic acid, topiramate, gabapentin)

Acute treatment

- Pain relievers (INNs: acetylsalicylic acid, paracetamol, diclofenac, flurbiprofen, ibuprofen, ketoprofen, ketorolac, metamizole sodium, naproxen, phenazone, tolfenamic acid, hydromorphine, meperidine, morphine, butorphanol).
- Triptans/Ditans (INNs: almotriptan, eletriptan, frovatriptan, naratriptan, rizatriptan, sumatriptan, zolmitriptan, lasmiditan).
- Combination triptan + NSAID
- Ergot alkaloids (INNs: ergotamine, dihydroergotamine).
- Neuroleptics (INNs: chlorpromazine, prochlorperazine, promethazine)
- Anti-emetic drugs (INNs: domperidone, metoclopramide, trimethobenzamide).

The descriptive statistics of Treatment patterns/Patients will be provided according to Section **Error! Reference source not found.**.

Treatment patterns/Patients with unmet need:

- Patients with different medications of interest
- Patients without therapy data
- Patients with acute therapy only
    -

5.6 Patient routing

Descriptive data will be provided according to Section **Error! Reference source not found.**.

The visits of patients will be summarised by:

- Inpatient visits, outpatient visits, emergency medical services;
    - Patients with inpatient visits only;
    - Patients with outpatient visits only;
    - Patients with emergency medical services only;
    - Patients with inpatient and outpatient visits;
    - Patients with emergency medical services and outpatient visits.
- Specialist visits (neurologist, cardiologist, ophthalmologist, endocrinologist, gastroenterologist, otolaryngologist, internist, surgeon, general practitioner).

Also, for these visits will be summarised by main disease profile and overall:

- Treatments and prescriptions given in the ED.
- Types and numbers of radiological examinations related to brain and cervical regions;
    - Patients with outpatient visits only;
    - Patients with emergency medical services only;
    - Patients with emergency medical services and outpatient visits.

To calculate the economic burden of migraines, the annual determination of doctor visits, ED visits, the number of prescriptions, and the count of radiological tests is required with these diagnosis codes. Therefore, all calculations will be done for each year separately.

5.7 Other

Other parameters will be summarised:

- Alcohol and tobacco use;
- Presence or absence of allergies;
- Number of patients who received Emgality, Kuzelva, Botox, or GON blocks from the finance card module's Charge section;
    - Number of applications;
- Number of neurology department visits in a year;
- Adverse events (if applicable);
- Complications.

Histograms of percentage and absolute values for categorial data will be performed by specified subgroups-variables and overall. For continues data will be presented the spaghetti plot with age category on abscissa axis and mean ± standard deviation by subgroups-variables and overall, on ordinate axis.

6. REFERENCES

1) ICH. (5 February 1998). ICH Harmonised Tripartite Guideline, Statistical Principles for Clinical Trials (E9) https://database.ich.org/sites/default/files/E9_Guideline.pdf
2) SOP-STAT-001 Statistical Principles
3) SOP-STAT-002 Development of Statistical Analysis Plan
4) ICH. (November 1995). "ICH Harmonised Tripartite Guideline, Structure and Content of Clinical Study Reports E3. https://database.ich.org/sites/default/files/E3_Guideline.pdf
5) Migren BaşAğrısı Tedavisi, Turkish Society of Neurology/Türk Nöroloji Derneği, https://noroloji.org.tr/html/file/335.pdf
6) Migraine Headache Treatment, Medically Reviewed by Murtaza Cassoobhoy, MD on February 09, 2023, https://www.webmd.com/migraines-headaches/migraine-treatments
7) de Vries, T., Villalón, C. M., & MaassenVanDenBrink, A. (2020). Pharmacological treatment of migraine: CGRP and 5-HT beyond the triptans. *Pharmacology & Therapeutics*, *211*, 107528.
