# Supplementary material for: Real-world evidence on migraine treatment in routine clinical practice: insights from the Türkiye migraine registry study using artificial intelligence algorithms
Source: Front Neurol. 2026 Aug 17;17:1848551. doi: 10.3389/fneur.2026.1848551 (PMC13526660; doi:10.3389/fneur.2026.1848551)
Supplement: Supplementary file 3 [file Data_Sheet_3.pdf]

Symptoms Reported by Patients During Migraine Attacks (N = 8,881)

Symptom	n	%*
Headache	8,881	100.0
Nausea	4,447	50.1
Photophobia	2,327	26.2
Phonophobia	1,694	19.1
Vomiting	1,428	16.1
Dizziness	1,415	15.9
Numbness	869	9.8
Weakness	628	7.1
Paresthesia	581	6.5
Sleeping disturbance	391	4.4
Insomnia	389	4.4
Impaired vision	320	3.6
Amnesia	319	3.6
Blurred vision	297	3.3
Spasm	288	3.2
Bruxism	280	3.2
Anxiety	231	2.6
Tremor	226	2.5
Dermatological symptoms	209	2.4
Abnormal voice hearing	204	2.3
Osmophobia	204	2.3
Psychological abnormality	201	2.3
Fainting	166	1.9
Sweating	127	1.4
Abnormal visual sensation	124	1.4
Neck pain	122	1.4
Impaired speech	114	1.3
Aura	108	1.2
Somnolence	83	0.9
Distractibility	81	0.9
Impaired hearing	81	0.9
Apnea	75	0.8
Allergy	72	0.8
Eye pain	72	0.8
Snoring	72	0.8

Symptom	n	%*
Fever	70	0.8
Bleeding	68	0.8
Cognitive impairment	62	0.7
Shortness of breath	62	0.7
Diarrhea	60	0.7
Back pain	53	0.6
Anorexia	41	0.5
Abnormal smelling	33	0.4
Stomach pain	29	0.3
Itching	19	0.2
Body pain	17	0.2
Urinary incontinence	14	0.2
Chest pain	12	0.1
Musculoskeletal pain	10	0.1
Hallucination	8	0.1
Jaw pain	5	<0.1
Hoarseness	2	<0.1
Shoulder pain	2	<0.1
Sore throat	2	<0.1
Depression	1	<0.1
Ear pain	1	<0.1
Epilepsy	1	<0.1
Groin pain	1	<0.1
Knee pain	1	<0.1
Stress	1	<0.1

N: Number of subjects

*Percentages are calculated using headache-positive patients (N=8,881) as the denominator. Multiple symptoms could be reported by the same patient; therefore, percentages do not sum to 100%.
