# Supplementary material for: Real-world evidence on migraine treatment in routine clinical practice: insights from the Türkiye migraine registry study using artificial intelligence algorithms
Source: Front Neurol. 2026 Aug 17;17:1848551. doi: 10.3389/fneur.2026.1848551 (PMC13526660; doi:10.3389/fneur.2026.1848551)
Supplement: Supplementary file 4 [file Supplementary_file_1.docx]

**Supplementary Table S1. Performance Metrics of the AI-Based Information Extraction Framework**

| Task | Precision (%) | Recall (%) | F1-score (%) | Validation Approach |
| --- | --- | --- | --- | --- |
| Named entity recognition (overall) | NR* | NR* | >95 | Validation dataset based on 573 manually annotated Turkish clinical free-text samples |
| Symptom extraction | 85.9 | 85.9 | 85.9 | Internal validation |
| Medication extraction | 97.5 | 96.4 | 97.0 | Internal validation |

AI: Artificial intelligence, NER: Named entity recognition, NR: Not reported.

**Footnote**

For the overall NER model, only the overall F1-score was available from the proprietary validation process. Precision and recall values were not separately available to the investigators. Confidence intervals were not available because performance metrics were generated as part of the proprietary internal validation framework developed by Traverse Health.
